# The established of a machine learning model for predicting the efficacy of adjuvant interferon alpha1b in patients with advanced melanoma

**DOI:** 10.3389/fimmu.2024.1495329

**Published:** 2024-11-12

**Authors:** Linhan Jiang, Ke Su, Jing Wang, Yitong Lin, Xianya Zhao, Hengxiang Zhang, Yu Liu

**Affiliations:** ^1^ Department of Dermatology, Xijing Hospital, Fourth Military Medical University, Xi’an, Shaanxi, China; ^2^ Department of Radiation Oncology, National Cancer Center/National Clinical Research Center for Cancer/Cancer Hospital, Chinese Academy of Medical Sciences and Peking Union Medical College, Beijing, China; ^3^ Department of Oncology, The Affiliated Hospital of Southwest Medical University, Luzhou, Sichuan, China

**Keywords:** immunotherapy, machine learning, melanoma, interferon-alpha, adjuvant therapy, prognostic factors

## Abstract

**Background:**

Interferon-alpha1b (IFN-α1b) has shown remarkable therapeutic potential as adjuvant therapy for melanoma. This study aimed to develop five machine learning models to evaluate the efficacy of postoperative IFN-α1b in patients with advanced melanoma.

**Methods:**

We retrospectively analyzed 113 patients with the American Joint Committee on Cancer (AJCC) stage III-IV melanoma who received postoperative IFN-α1b therapy between July 2009 and February 2024. Recurrence-free survival (RFS) and overall survival (OS) were assessed using Kaplan-Meier analysis. Five machine learning models (Decision Tree, Cox Proportional Hazards, Random Forest, Support Vector Machine, and LASSO regression) were developed and compared for their capacity to predict the outcomes of patients. Model performance was evaluated using concordance index (C-index), time-dependent receiver operating characteristic (ROC) curves, and decision curve analysis.

**Results:**

The 1-year, 2-year, and 3-year RFS rates were 71.10%, 43.10%, and 31.10%, respectively. For OS, the 1-year, 2-year, and 3-year OS rates were 99.10%, 82.30%, and 75.00%, respectively. The Decision Tree (DT) model demonstrated superior predictive performance with the highest C-index of 0.792. Time-dependent ROC analysis for predicting 1-, 2-, and 3-year RFS based on the DT model is 0.77, 0.79 and 0.76, respectively. Serum albumin emerged as the important predictor of RFS.

**Conclusions:**

Our study demonstrates the considerable efficacy DT model for predicting the efficacy of adjuvant IFN-α1b in patients with advanced melanoma. Serum albumin was identified as a key predictive factor of the treatment efficacy.

## Introduction

The global incidence of melanoma has been increasing gradually, presenting a considerable public health challenge (1, 2). The incidence rate of melanoma vary geographically, with the highest rates observed in Australia and New Zealand, followed by North America and Europe (3). Notably, in Asian populations, although the overall incidence remains lower, melanoma frequently manifests as acral or mucosal subtypes, which are associated with poorer prognosis (4).

Surgical excision is the most prevalent treatment modality for melanoma. However, postoperative recurrence rates remain substantial (5). This high recurrence risk necessitates the exploration of effective adjuvant therapies to improve long-term outcomes. The challenge is particularly pronounced in Chinese populations, where acral and mucosal melanoma subtypes are more prevalent (6, 7). These subtypes exhibit lower response rates to immune checkpoint inhibitors like anti-PD-1 and anti-CTLA4 antibodies (7, 8), resulting in inferior clinical prognosis for patients with advanced melanoma. Consequently, there is a pressing need to develop alternative therapeutic method that can improve clinical outcomes, particularly for melanoma in Chinese populations.

Among the adjuvant therapeutic strategies, the interferon-α1b (IFN-α1b) has revealed promising treatment effect. As a member of the interferon α family, IFN-α1b exhibits relatively favorable tolerability, eliciting anti-tumor responses and potentially playing a central role in innate immunity, thereby providing a rationale for its clinical application in oncology (9–12). Recently, our team has demonstrated the promising potential of human IFN-α1b in melanoma treatment. A retrospective study by Shi et al. showed that IFN-α1b monotherapy exhibited favorable outcomes in unresectable stage IV melanoma patients, resulting in a median overall survival (mOS) of 14.1 months (13). Subsequently, Gao et al. reported the efficacy of adjuvant IFN-α1b in resected stage IIIB or IIIC melanoma. This study revealed promising recurrence-free survival rates of 75.4%, 47.4%, and 37.2% at 12, 24, and 36 months, respectively. Moreover, the overall survival rates were impressive, with 100%, 81.9%, and 71.5% at the same time points (14). Despite these encouraging results, several important questions remain. The optimal criteria for patient selection and the long-term efficacy of IFN-α1b as an adjuvant therapy remain to be fully elucidated. Furthermore, given the heterogeneity of melanoma and the variability of treatment responses, there is a pressing need for more sophisticated prognostic tools to guide treatment decisions and predict outcomes in patients receiving adjuvant IFN-α1b therapy.

In recent years, machine learning has emerged as a promising tool in oncology, providing novel opportunities for predicting treatment outcomes and personalizing patient care (15). By analyzing intricate patterns within large datasets, machine learning algorithms have the potential to identify subtle prognostic factors and treatment response indicators that may not be discernible through traditional statistical methods (16). This approach is particularly promising in the context of melanoma, where the heterogeneity of the disease and the variability in treatment responses present substantial challenges to clinical decision-making (17). Based on these considerations, our study aims to evaluate the efficacy of postoperative IFN-α1b in melanoma patients. More importantly, we want to develop and validate machine learning models for assessing prognosis of these patients, by integrating clinical data with advanced machine learning methodologies, so as to optimize melanoma management and improve the outcome of patients with melanoma, especially in Chinese populations.

## Materials and methods

### Study design and patient population

Profiles of patients diagnosed with melanoma (the American Joint Committee on Cancer (AJCC) 8th Edition) based on clinical and histological confirmation from July 1st, 2009 to February 29th, 2024 in the Department of Dermatology, Xijing Hospital were reviewed. This study was conducted in accordance with the Declaration of Helsinki and was approved by the Internal Review Board of Air Force Military Medical University (KY20242167-C-1).

Inclusion criteria were: (1) underwent surgical treatment; (2) stages III-IV; (3) treatment with postoperative IFN-α1b therapy for a minimum duration of one month; and (4) complete clinical, pathological, and follow-up data. Exclusion criteria included: (1) patients with unknown primary sites; (2) age <18 years; (3) prior systemic therapy for melanoma; (4) concurrent malignancy; and (5) incomplete medical records.

### Data collection and outcome measures

Patient data were extracted from electronic medical records and pathology reports. Collected variables included demographic information (age, sex), clinical characteristics (TNM stage, Eastern Cooperative Oncology Group (ECOG) performance status at last follow-up), and pathological features. Laboratory data encompassed complete blood count parameters (white blood cell count, neutrophil count, lymphocyte count, hemoglobin level, platelet count) and liver function tests (ALT, AST, albumin, alkaline phosphatase). Hepatitis B virus (HBV) status was also recorded. Treatment details included surgical information (type of surgery, whether the primary lesion was resected), and the details of IFN-α1b adjuvant therapy. Outcome measures comprised recurrence-free survival (RFS), overall survival (OS), recurrence information, and survival status.

Follow-up assessments, including physical examination, complete blood count, and serum biochemical tests, were performed prior to therapy initiation and at 3-month intervals thereafter. Imaging studies, including ultrasound evaluation of all lymph nodes and CT scans of the chest, abdomen, and pelvis, were conducted at baseline and every 3 months to assess the status of recurrence and distant metastases. Besides, recurrence or metastatic lesions were confirmed through histopathological examination when possible.

The primary endpoint was RFS, defined as the time from diagnosis to the date of initial recurrence (local, regional, or distant metastasis) or death from any cause. The secondary endpoint was OS, defined as the time from diagnosis to death from any cause. Patients without events were censored at the date of last follow-up or September 6th, 2024, whichever came first.

### Machine learning algorithms

The dataset was then randomly split into training and validation cohorts at 6:4 ratio. Five machine learning models (Decision Tree (DT), Cox proportional-hazards model, Least Absolute Shrinkage and Selection Operator (LASSO), Random Forest (RF), and Support Vector Machine (SVM)) were developed to assess the survival outcomes.

Hyperparameter tuning was conducted using grid search with 5-fold cross-validation on the training set. Model performance was evaluated using the concordance index (C-index) and time-dependent receiver operating characteristic (ROC) curves. The area under the ROC curve (AUC) at 1, 2, and 3 years was calculated to assess the models’ discriminative ability over time. Additionally, decision curve analysis (DCA) was employed to evaluate the clinical utility of the best-performing model across a range of threshold probabilities.

To interpret the model, we employed SurvSHAP, which calculates the average SHAP (SHapley Additive exPlanations) value for each feature across all samples. Time-dependent variable importance bar plots were utilized to determine significant features for 1-, 2-, and 3-year survival. Furthermore, partial dependence plots (PDPs) were employed to show how variations in feature values affect the predicted outcomes.

### Statistical analysis

Categorical variables were presented as frequencies and percentages, while continuous variables were expressed as median and interquartile range (IQR) or mean ± standard deviation (SD) as appropriate. Comparisons between groups were performed using the chi-square test or Fisher’s exact test for categorical variables and the Mann-Whitney U test or t-test for continuous variables, as appropriate. RFS and OS were evaluated and depicted using the Kaplan-Meier method with the log-rank test. All statistical analyses and machine learning model development were performed using R software version 4.1.0 (R Foundation for Statistical Computing, Vienna, Austria). A two-sided P < 0.05 was considered statistically significant.

## Results

### Patient characteristics

We retrospectively analyzed 113 melanoma patients who received IFN-α1b adjuvant therapy. The baseline characteristics are summarized in **Table 1**. These patients were with mean age of 57.7 ± 12.7 years, and the majority (85.0%, n=96) at AJCC stage III. Among all these cases, acral melanoma was the predominant subtype (64.6%, n=73), followed by cutaneous (25.7%, n=29) and mucosal (9.73%, n=11) melanoma. High-dose IFN-α1b (≥600 μg) was administered to 61.1% (n=69) of patients. Comorbidities were present in a subset, with diabetes mellitus and hypertension observed in 11.5% (n=13) and 28.3% (n=32) of patients, respectively.

**Table 1:** Baseline characteristics of patients.

Variable	Overall, N=113
Stage	
III	96 (85.0%)
IV	17 (15.0%)
Age, years, mean ± SD	57.7 ± 12.7
< 60	54 (47.8%)
≥ 60	59 (52.2%)
Sex	
Female	60 (53.1%)
Male	53 (46.9%)
Primary site	
Acral	73 (64.6%)
Cutaneous	29 (25.7%)
Mucosal	11 (9.73%)
Diabetes mellitus	
No	100 (88.5%)
Yes	13 (11.5%)
Hypertension	
No	81 (71.7%)
Yes	32 (28.3%)
Ki-67	
< 20%	27 (23.9%)
≥ 20%	63 (55.8%)
Uknow	23 (20.4%)
Interferon dose, μg	
< 600	44 (38.9%)
≥ 600	69 (61.1%)
ECOG	
0	98 (86.7%)
≥ 1	15 (13.3%)
HBV	
No	92 (81.4%)
Yes	21 (18.6%)
WBC, * 10^9^/L	5.97 ± 1.91
NEU, * 10^9^/L	3.75 ± 1.69
LYM, * 10^9^/L	2.26 ± 1.76
Hb, g/L	153 ± 47.4
PLT, 100 * 10^9^/L	207 ± 64.2
ALT, U/L	22.3 ± 12.2
AST, U/L	21.7 ± 10.4
ALB, g/L	44.1 ± 4.20
ALP, U/L	84.4 ± 30.3

ALB, Albumin; ALP, Alkaline Phosphatase; ALT, Alanine Aminotransferase; AST, Aspartate Aminotransferase; ECOG, Eastern Cooperative Oncology Group; Hb, Hemoglobin; HBV, Hepatitis B Virus; IFN-α1b, Interferon-alpha1b; LYM, Lymphocyte; NEU, Neutrophil; PLT, Platelet; SD, Standard Deviation; WBC, White Blood Cell.

### Survival analysis

**Figure 1:**
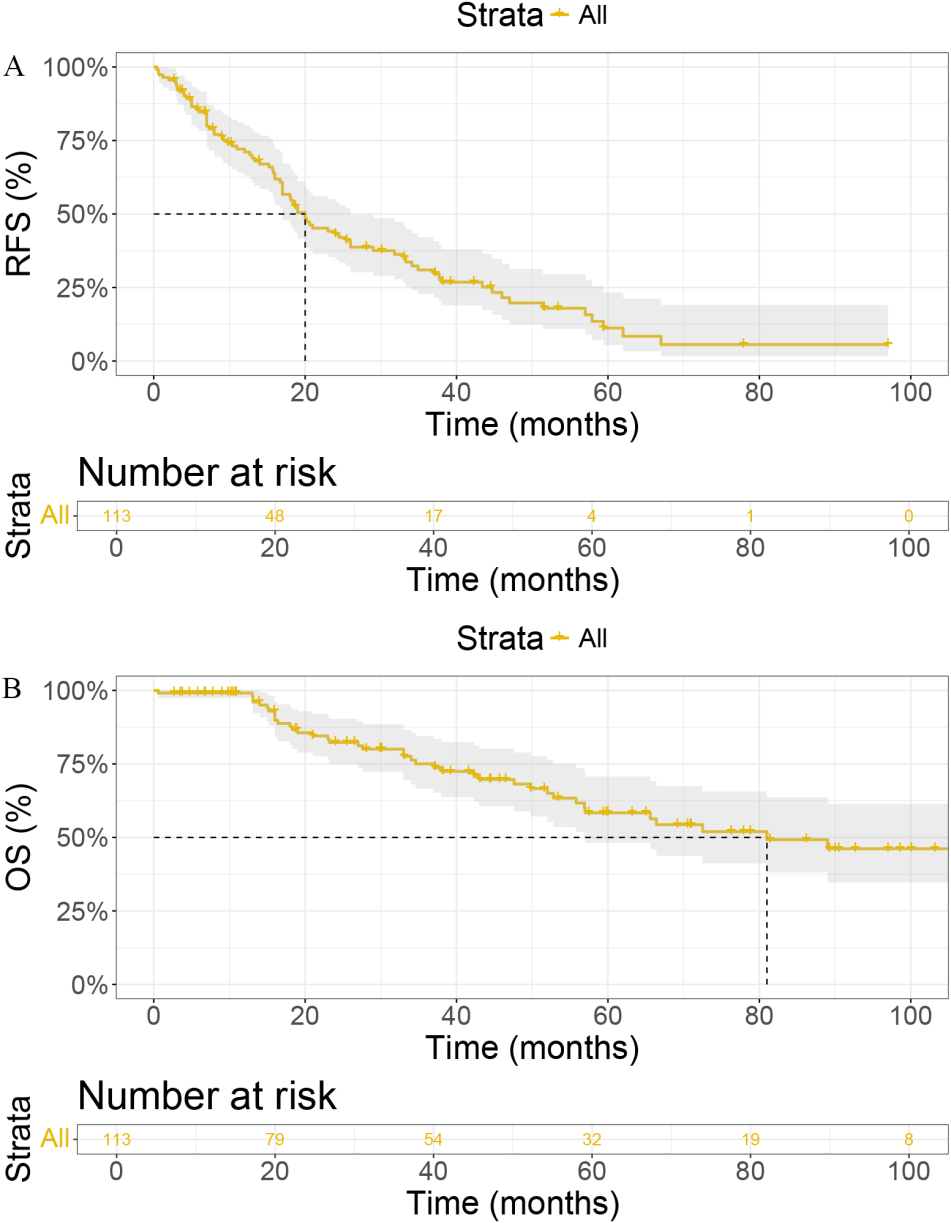
Kaplan-Meier curves for **(A)** Recurrence-Free Survival (RFS) and **(B)** Overall Survival (OS) in patients. RFS, Recurrence-Free Survival; OS, Overall Survival; IFN-α1b, Interferon-alpha1b; CI, Confidence Interval.

We conducted Kaplan-Meier analyses to evaluate recurrence-free survival (RFS) and overall survival (OS) in our cohort (**Figure 1**). The median RFS was 20 months (95% CI: [17, 26]), with 1-year, 2-year, and 3-year RFS rates of 71.10%, 43.10%, and 31.10%, respectively (**Figure 1A**). For OS, the median was 81 months (95% CI: [56.9, NA]), with 1-year, 2-year, and 3-year OS rates of 99.10%, 82.30%, and 75.00%, respectively (**Figure 1B**).

### Machine learning model evaluation

**Figure 2:**
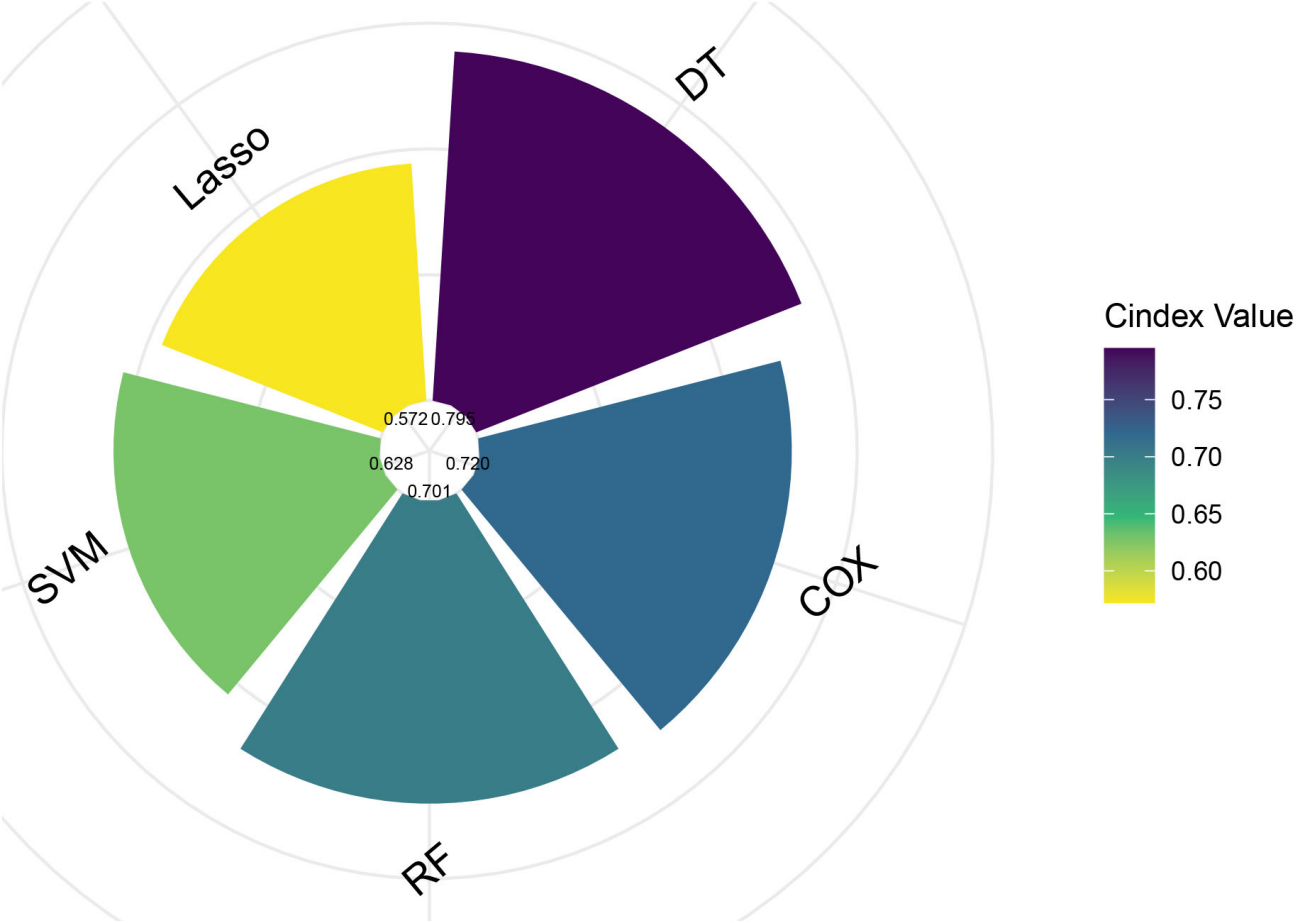
Comparison of C-index values for five different predictive models. COX, Cox Proportional Hazards; DT, Decision Tree; Lasso, Least Absolute Shrinkage and Selection Operator; RF, Random Forest; SVM, Support Vector Machine.

In the training set, we developed and compared five machine learning models to predict melanoma patient outcomes: Decision Tree (DT), Cox Proportional Hazards (COX), Random Forest (RF), Support Vector Machine (SVM), and Lasso regression. Model performance was initially evaluated using the C-index (**Figure 2**). In the validation set, the DT model demonstrated superior predictive performance with the highest C-index of 0.792, followed by COX (0.720), RF (0.701), SVM (0.628), and Lasso regression (0.572).

**Figure 3:**
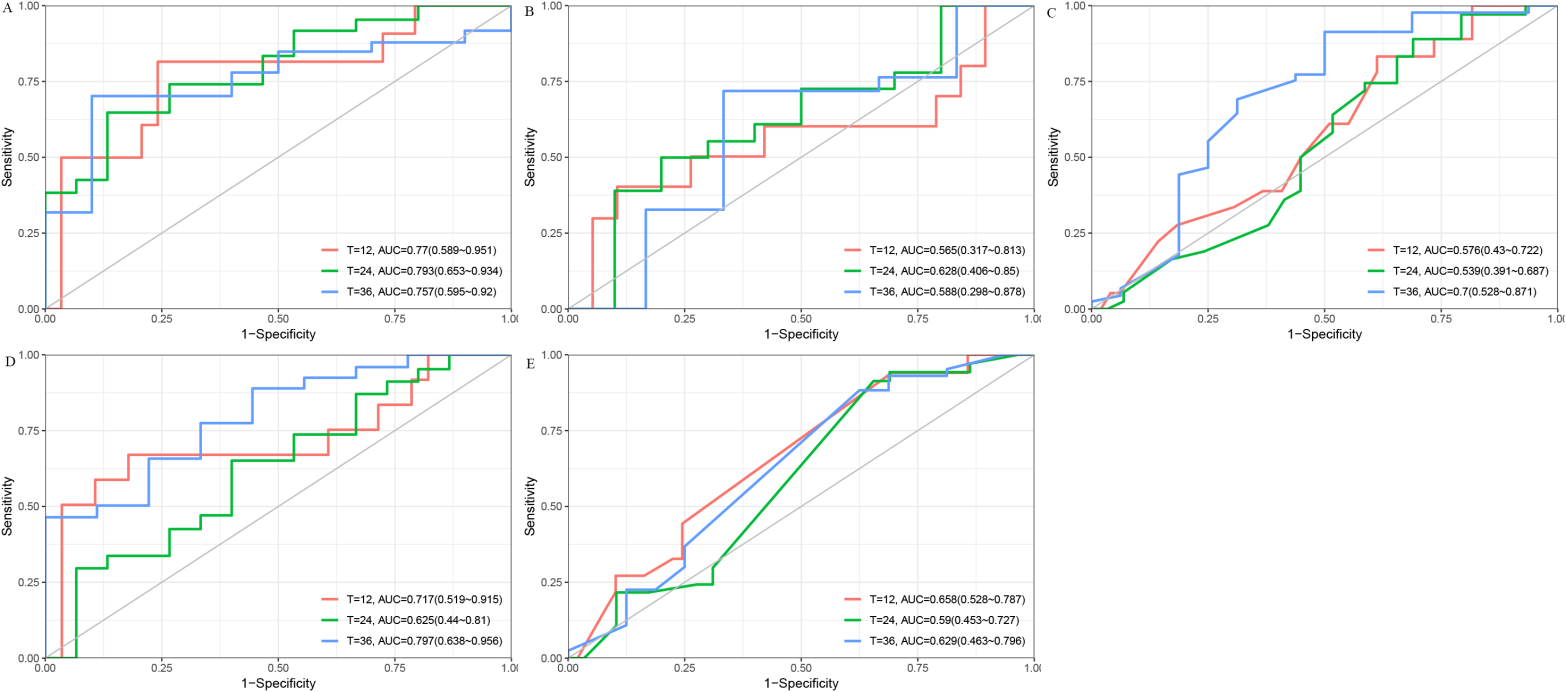
The ROC curves for predicting 1-, 2-, and 3-year RFS using five different models. **(A)** DT model. **(B)** Cox Proportional Hazards model. **(C)** Lasso model. **(D)** RF model. **(E)** SVM model. AUC, Area Under the Curve; Cox, Cox Proportional Hazards; DT, Decision Tree; Lasso, Least Absolute Shrinkage and Selection Operator; RF, Random Forest; ROC, Receiver Operating Characteristic; RFS, Recurrence-Free Survival; SVM, Support Vector Machine.

**Figure 4:**
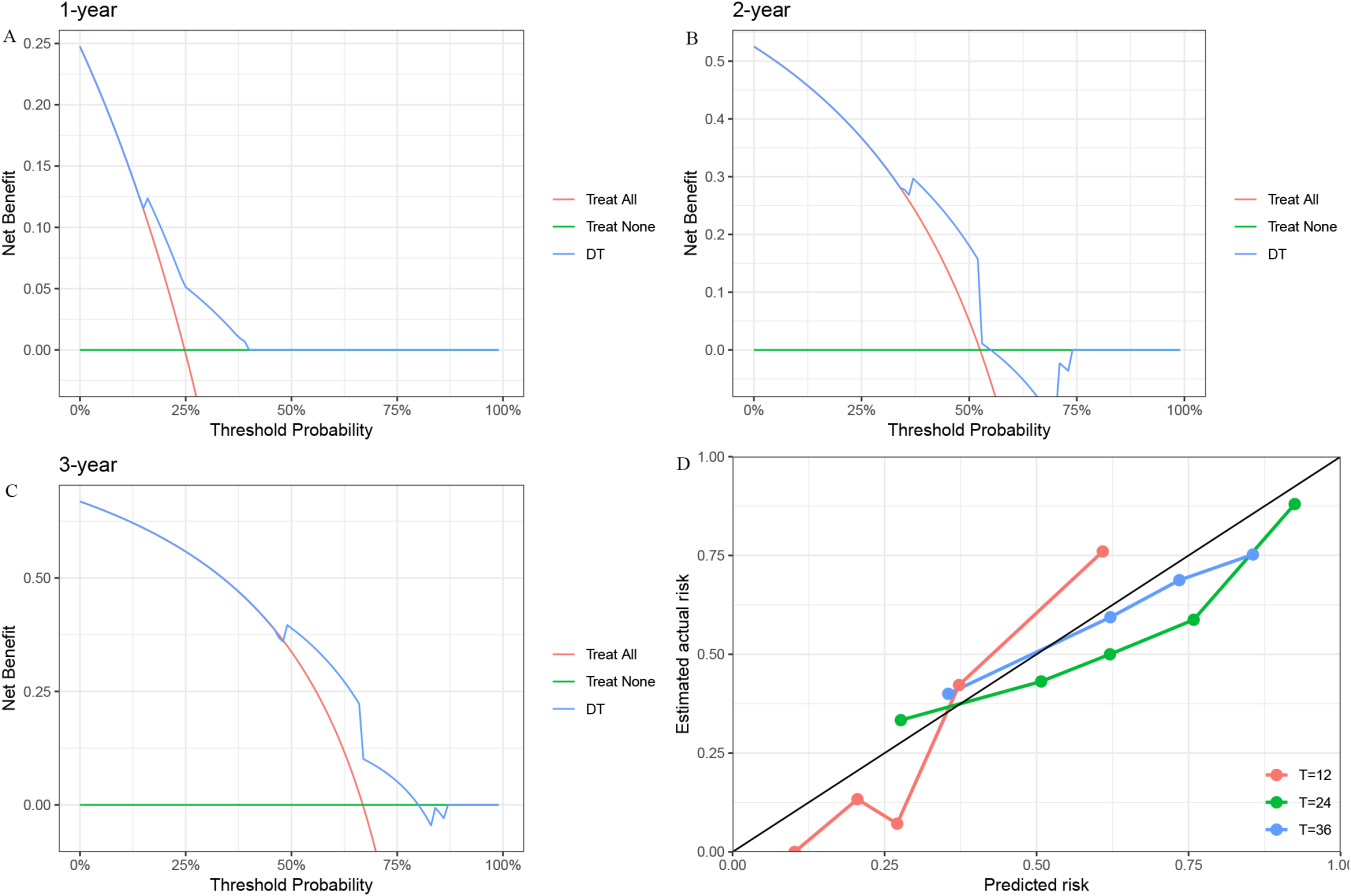
The DCA and Calibration Curves for the DT model in predicting RFS. **(A)** 1-year RFS. **(B)** 2-year RFS. **(C)** 3-year RFS. **(D)** Calibration Curve. DCA, Decision Curve Analysis; DT, Decision Tree; RFS, Recurrence-Free Survival.

To further assess predictive performance, we conducted time-dependent ROC analysis for RFS at 1, 2, and 3 years (**Figures 3A–E**). The DT model consistently outperformed other models across all time points, with 1-, 2-, and 3-year RFS of 0.77 (95% CI: [0.59, 0.95]), 0.79 (95% CI: [0.65, 0.93]), and 0.76 (95% CI: [0.60, 0.92]), respectively (**Figure 3A**). Furthermore, the DCA (**Figures 4A–C**) and calibration curves (**Figure 4D**) for predicting 1-, 2-, and 3-year RFS based on the DT model all demonstrated good predictive performance.

### Feature analysis

To identify key predictors of RFS in melanoma patients receiving IFN-α1b adjuvant therapy, we performed the time-dependent feature importance analysis, including tumor characteristics (site and stage), patient demographics (age and sex), comorbidities (diabetes mellitus, hypertension and hepatitis B virus) and various laboratory indices (neutrophil (NEU), alanine aminotransferase, aspartate aminotransferase, lymphocyte, hemoglobin (Hb), albumin (ALB), white blood cell, platelet and alkaline phosphatase) (**Supplementary Figure 1**). The analysis revealed that ALB emerged as the most significant predictor of RFS, maintaining its top ranking across all time points.

To further elucidate the complex relationships between key laboratory parameters and RFS, we conducted partial dependence survival analyses (**Supplementary Figure 2**). These analyses revealed non-linear associations, with higher values of NEU, ALB, and Hb consistently linked to improved RFS outcomes.

Complementing these findings, the box plots based on Shapley values (**Supplementary Figures 3**, **4**) provided granular insights into the contribution of various laboratory parameters to RFS prediction at 12-, 24-, and 36-months post-treatment. Consistent with our previous findings, ALB and NEU emerged as key predictors, with higher values associated with improved RFS across all time points.

## Discussion

In this retrospective study, we demonstrated the efficacy of adjuvant IFN-α1b therapy in improving clinical outcomes for patients with resected stage III-IV melanoma. Our analysis revealed a median RFS of 20 months and a median OS of 81 months, underscoring the potential of IFN-α1b in the management of advanced melanoma. To further refine our understanding and prediction of treatment efficacy, we developed and compared five machine learning models. Among these, the DT model demonstrated superior performance in predicting individual patient responses to IFN-α1b therapy. This innovative approach not only validated the overall efficacy of the treatment but also provided a novel tool for personalized treatment decisions in melanoma management.

The efficacy of IFN-α1b in melanoma treatment can be attributed to its multifaceted biological effects. Primarily, IFN-α1b exerts direct antiproliferative effects on tumor cells by inducing cell cycle arrest and apoptosis (18). Additionally, it enhances the immune response against melanoma cells through multiple mechanisms: activating natural killer cells, upregulating the expression of major histocompatibility complex (MHC) class I molecules, and promoting the differentiation of dendritic cells (19–21). Recent studies have also highlighted the role of IFN-α1b in modulating the tumor microenvironment, including the inhibition of angiogenesis and the promotion of a more immunogenic tumor phenotype (18, 19). These mechanisms contribute to the observed clinical benefits in our study, reinforcing the rationale for IFN-α1b as an adjuvant therapy in advanced melanoma.

Our findings align with and extend previous studies on interferon therapy in melanom. A meta-analysis by Ives et al. demonstrated a significant improvement in RFS with interferon therapy, consistent with our results (22). However, our study uniquely contributes to the field by employing machine learning techniques to predict individual patient responses, an approach not previously applied in this context. While recent research has focused on alternative immunotherapies like PD-1 inhibitors (23), our study reaffirms the value of IFN-α1b, particularly in melanoma patients in Chinese population who may exhibit lower response rates to immune checkpoint inhibitors. Moreover, our machine learning approach offers a novel strategy for patient stratification, potentially optimizing the use of IFN-α1b and may address the ongoing challenge of treatment selection in advanced melanoma.

The application of machine learning, particularly the DT model, represents a significant methodological advancement in predicting IFN-α1b treatment efficacy. The DT model offers several advantages over traditional statistical methods in this context. Firstly, they can capture non-linear relationships and complex interactions between variables, which are often present in biological systems (24). Secondly, the DT model provides easily interpretable results, allowing clinicians to understand the decision-making process, a crucial factor in medical applications (25). The hierarchical structure of the DT model also aligns well with clinical decision-making processes, making the model’s predictions more intuitive (26). Furthermore, DTs are robust to outliers and missing data, common challenges in clinical datasets (27). In our study, the DT model outperformed traditional logistic regression in both accuracy and area under the receiver operating characteristic curve (AUC-ROC), demonstrating its superior predictive capability in this complex clinical scenario.

Our machine learning model identified serum albumin levels as the most significant predictor of treatment response and prognosis in melanoma patients receiving IFN-α1b therapy. This finding aligns with emerging evidence on the crucial role of albumin in cancer biology and treatment outcomes. Albumin serves as a key indicator of nutritional status and overall health, factors known to influence cancer prognosis (28). A large-scale study by Gupta et al. found that pretreatment serum albumin levels were independently associated with overall survival in cancer patients across multiple tumor types (28). In the study conducted by Xie et al., the overall survival was significantly higher in the group with elevated albumin levels than in the lower albumin levels (29). Moreover, recent studies have elucidated albumin’s direct effects on tumor biology. Serum albumin has been shown to modulate the tumor microenvironment by influencing oxidative stress and inflammatory responses (30). It also plays a role in drug transport and metabolism, potentially affecting the pharmacokinetics and efficacy of IFN-α1b (31). Overall, serum albumin levels may serve as a biomarker reflecting the inflammatory status and nutritional state of patients. Low albumin levels could indicate a heightened inflammatory response or malnutrition, both of which are known to adversely affect melanoma prognosis. These multifaceted functions of albumin underscore its importance as a predictive biomarker in our model and suggest potential avenues for therapeutic interventions aimed at optimizing treatment outcomes in melanoma patients.

Despite the promising results, our study has several limitations that warrant consideration. Firstly, the retrospective, single-center design and non-random selection of patients introduce potential biases and limit the generalizability of our findings. Selection bias may have influenced patient inclusion, potentially affecting the observed treatment outcomes. The completeness and accuracy of clinical records may introduce errors that could affect the validity of our findings. What’s more, the single-center nature of the study also raises questions about the applicability of our machine learning model to diverse patient populations and clinical settings (32). Additionally, while our model showed good predictive performance, the relatively small sample size may have limited its ability to capture rarer prognostic factors or subgroup effects. The DT model demonstrated superior performance, likely due to the dataset’s characteristics, which may be more conducive to simpler models. Complex models, while potentially more powerful, risk overfitting, particularly in datasets with limited sample sizes. Future studies should explore the balance between model complexity and generalizability. Lastly, the evolving landscape of melanoma treatment, with the introduction of targeted therapies and novel immunotherapies, may impact the long-term relevance of our findings focused on IFN-α1b. Future multi-center, prospective studies with larger cohorts are needed to validate and refine our predictive model.

In conclusion, our study demonstrates the efficacy of IFN-α1b adjuvant therapy in melanoma and develops the DT model, which offers a promising tool for personalized risk assessment. The identification of serum albumin as a key predictive factor offers new insights into the biological mechanisms underlying treatment efficacy.

## Data Availability

The original contributions presented in the study are included in the article/**Supplementary Material**. Further inquiries can be directed to the corresponding author.

## Supplementary material

Supplementary Figure 1 The time-dependent feature for predicting recurrence-free survival. ALB, Albumin; ALT, Alanine Aminotransferase; ALP, Alkaline Phosphatase; AST, Aspartate Aminotransferase; ECOG, Eastern Cooperative Oncology Group; Hb, Hemoglobin; HBV, Hepatitis B Virus; IFN-α1b, Interferon-alpha1b; LYM, Lymphocyte; NEU, Neutrophil; PLT, Platelet; WBC, White Blood Cell.

Supplementary Figure 2 The partial dependence survival profiles for NEU, ALB, and Hb in relation to RFS. ALB, Albumin; Hb, Hemoglobin; IFN-α1b, Interferon-alpha1b; NEU, Neutrophil; RFS, Recurrence-Free Survival.

Supplementary Figure 3 The Shapley values for laboratory parameters at 12, 24, and 36 months predicting RFS. ALB, Albumin; ALP, Alkaline Phosphatase; ALT, Alanine Aminotransferase; AST, Aspartate Aminotransferase; Hb, Hemoglobin; LYM, Lymphocyte; NEU, Neutrophil; PLT, Platelet; RFS, Recurrence-Free Survival; WBC, White Blood Cell.

Supplementary Figure 4 The Shapley values for clinical features predicting RFS at 12, 24, and 36 months. ECOG: Eastern Cooperative Oncology Group performance status; HBV: Hepatitis B Virus.
