# Supplementary figures and images for: The established of a machine learning model for predicting the efficacy of adjuvant interferon alpha1b in patients with advanced melanoma
Source: Front Immunol. 2024 Nov 12;15:1495329. doi: 10.3389/fimmu.2024.1495329 (PMC11588685; doi:10.3389/fimmu.2024.1495329)

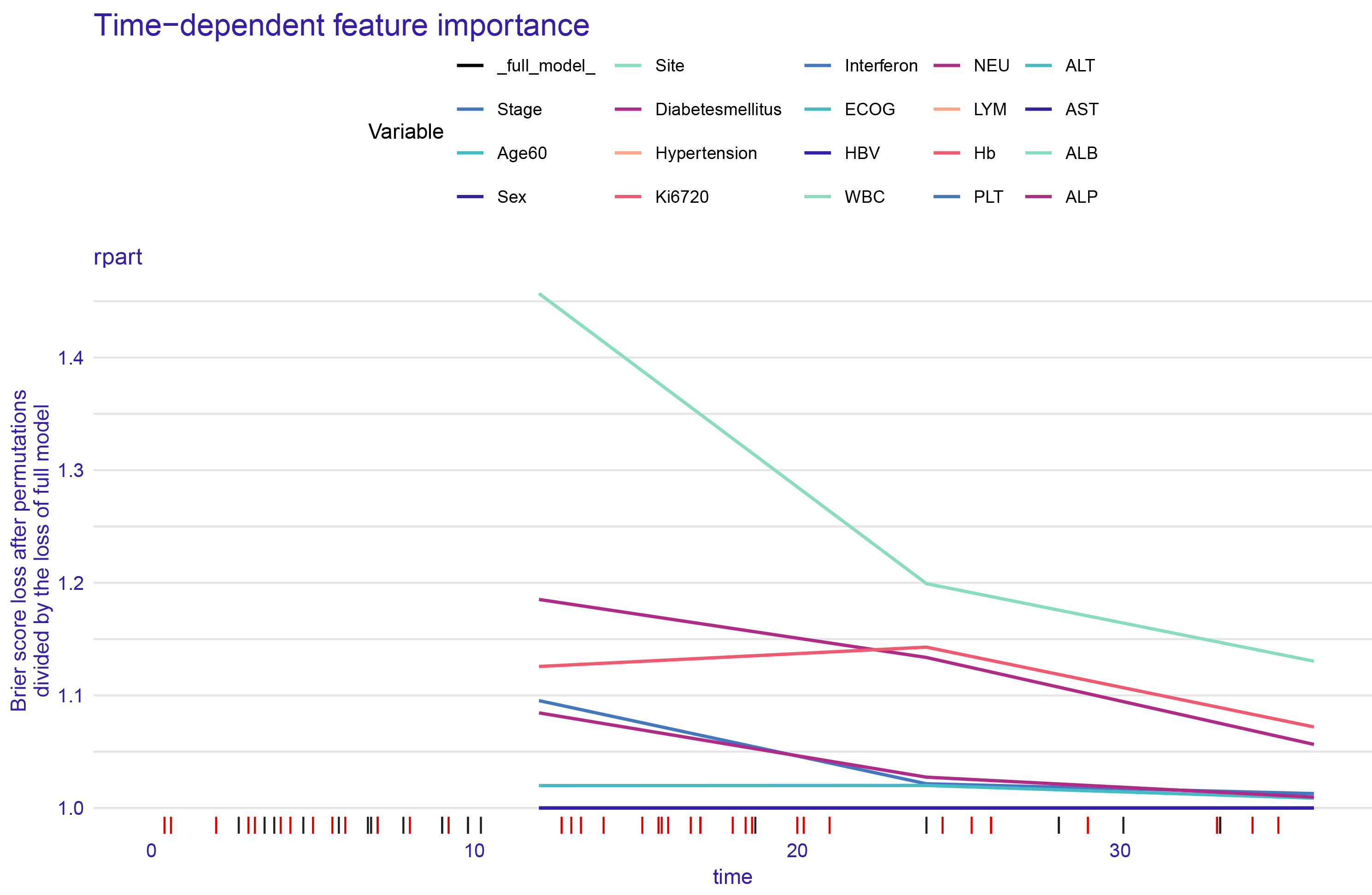

Supplement: Supplementary Figure 1 — The time-dependent feature for predicting recurrence-free survival. ALB, Albumin; ALT, Alanine Aminotransferase; ALP, Alkaline Phosphatase; AST, Aspartate Aminotransferase; ECOG, Eastern Cooperative Oncology Group; Hb, Hemoglobin; HBV, Hepatitis B Virus; IFN-α1b, Interferon-alpha1b; LYM, Lymphocyte; NEU, Neutrophil; PLT, Platelet; WBC, White Blood Cell. [file Image1.tif]

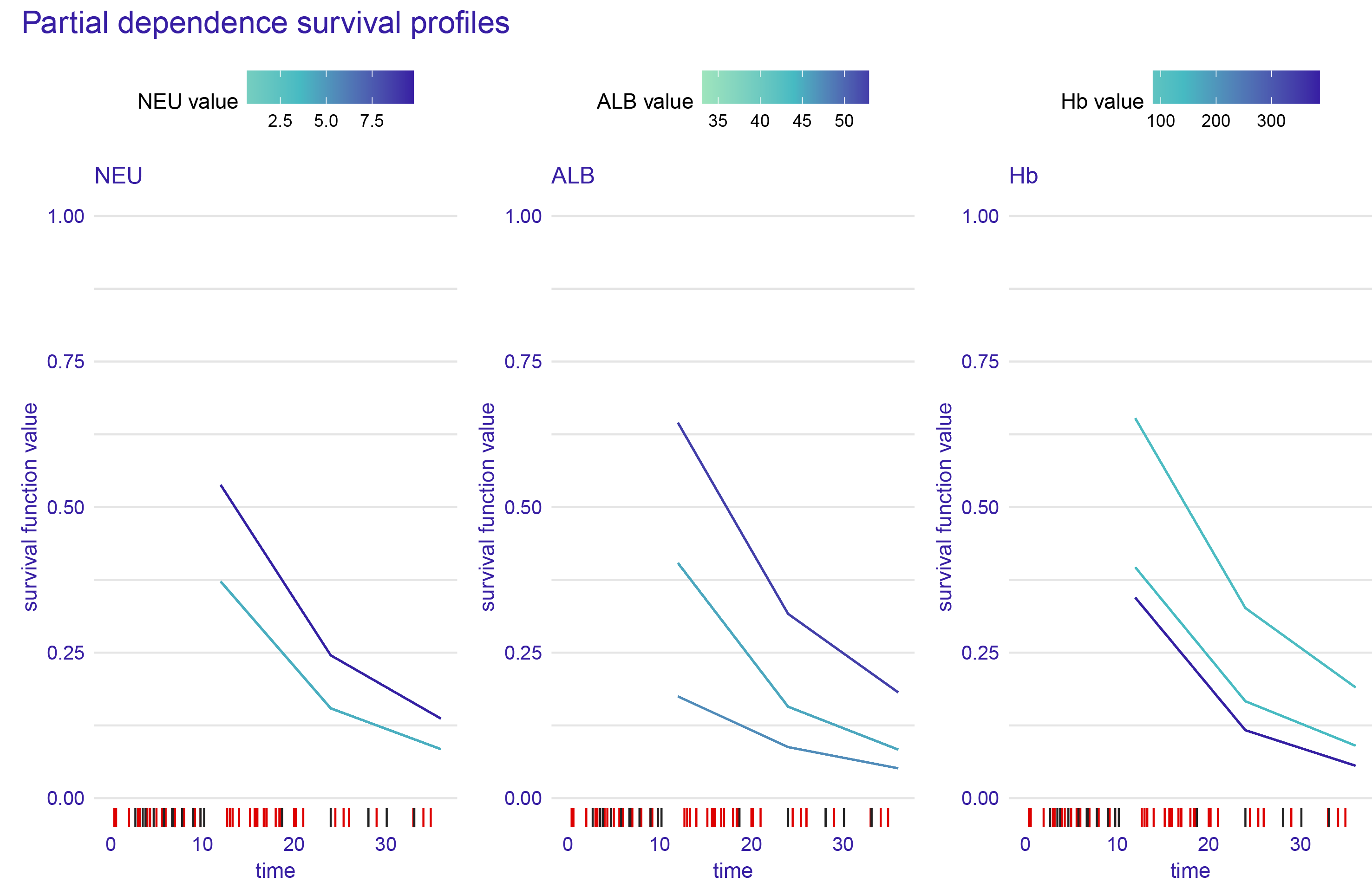

Supplement: Supplementary Figure 2 — The partial dependence survival profiles for NEU, ALB, and Hb in relation to RFS. ALB, Albumin; Hb, Hemoglobin; IFN-α1b, Interferon-alpha1b; NEU, Neutrophil; RFS, Recurrence-Free Survival. [file Image2.tif]

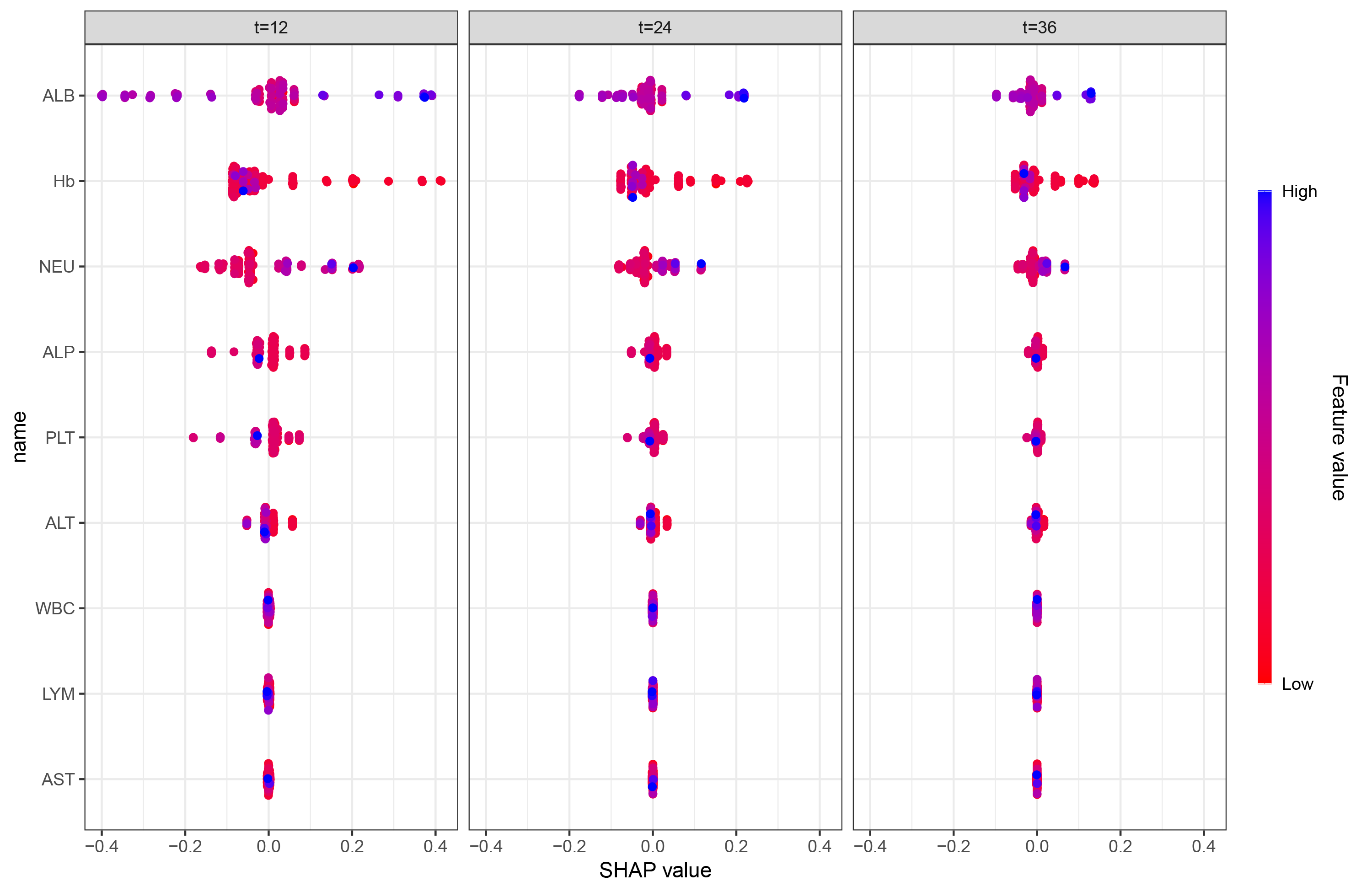

Supplement: Supplementary Figure 3 — The Shapley values for laboratory parameters at 12, 24, and 36 months predicting RFS. ALB, Albumin; ALP, Alkaline Phosphatase; ALT, Alanine Aminotransferase; AST, Aspartate Aminotransferase; Hb, Hemoglobin; LYM, Lymphocyte; NEU, Neutrophil; PLT, Platelet; RFS, Recurrence-Free Survival; WBC, White Blood Cell. [file Image3.tif]

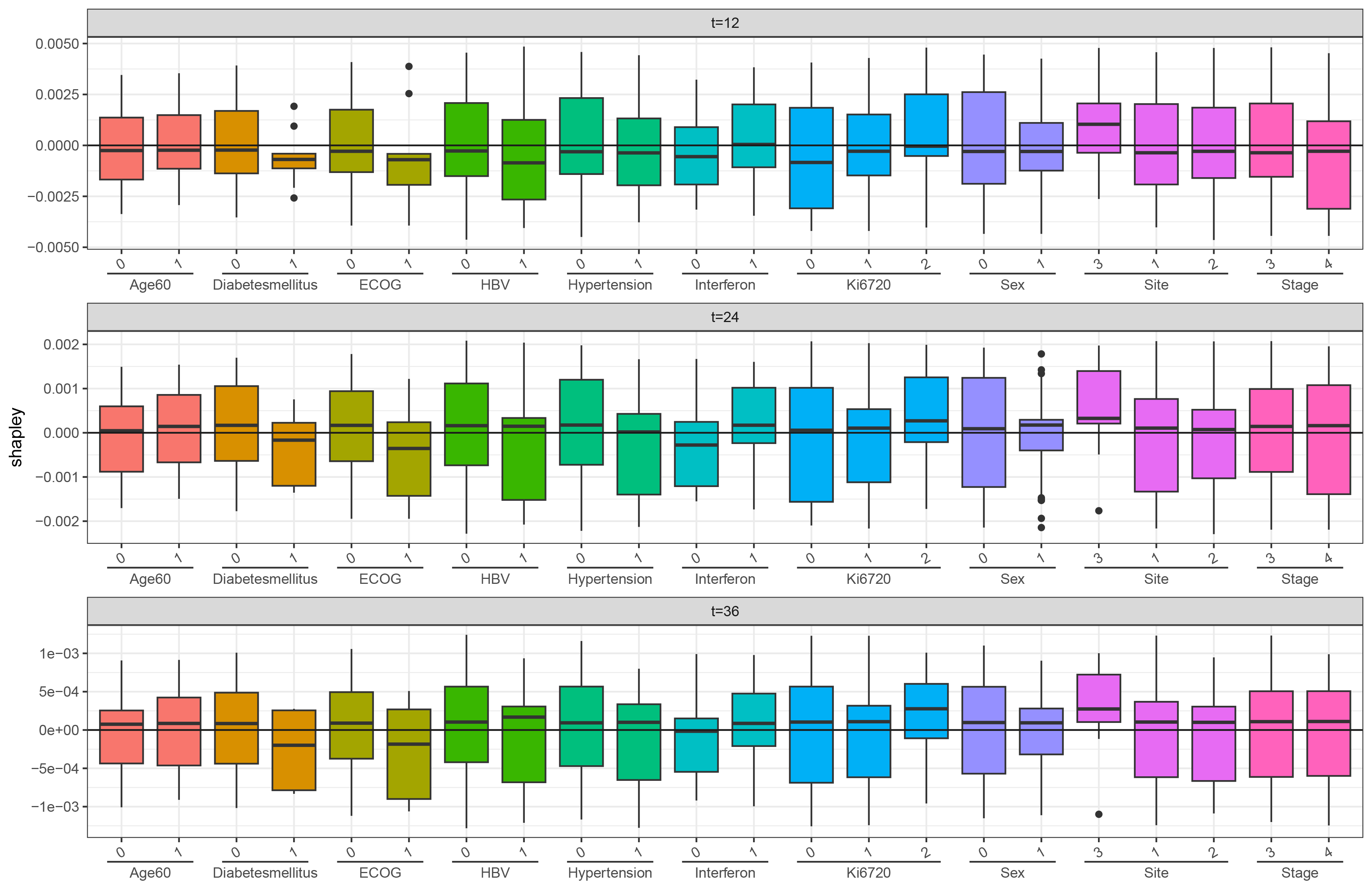

Supplement: Supplementary Figure 4 — The Shapley values for clinical features predicting RFS at 12, 24, and 36 months. ECOG: Eastern Cooperative Oncology Group performance status; HBV: Hepatitis B Virus. [file Image4.tif]
